# A Glimpse of Research Trends and Frontiers in the Etiology of Premature Ovarian Insufficiency *via* Bibliometric Analysis

**DOI:** 10.2174/0118715303313887240624071238

**Published:** 2024-06-25

**Authors:** Duan Li, Yingxue Liu, Yameng Hui, Bing Li, Cuifang Hao

**Affiliations:** 1 Centre for Reproductive Medicine, Women and Children’s Hospital, Qingdao University, Qingdao, China;; 2 Branch of Shandong Provincial Clinical Research Center for Reproductive Health, Qingdao, China;; 3 College of Medicine, Qingdao University, Qingdao, China;; 4 Department of Genetics and Cell Biology, Basic Medical College, Qingdao University, Qingdao, China;; 5 Qingdao Central Hospital, University of Health and Rehabilitation Sciences, Qingdao, China

**Keywords:** Premature ovarian insufficiency, pathogenesis, etiology, bibliometric analysis, VOSviewer, etiology research

## Abstract

**Introduction:**

Premature Ovarian Insufficiency (POI) is the most common reproductive aging disorder in women of reproductive age, which is characterized by decreased ovarian function in women before the age of 40. Etiology research of POI has garnered interest and attention from scholars worldwide over the past decades.

**Methods:**

However, to the best of our knowledge, no comprehensive survey with bibliometric analysis has been conducted yet on the research trends of POI etiology. This article aimed to analyze current scientific findings on the etiology of POI, offering innovative ideas for further research. Research articles on the etiology of POI from 1994 to 2023 were collected from the Web of Science Core Collection. A total of 456 research articles were included, and the total number of publications increased annually. We used VOSviewer and bibliometric.com to analyze the keywords, terms, institution, publication country/region, author name, publication journal, and the sum of times the articles have been cited.

**Results:**

This study has shown that a research hotspot is the genetic etiology of POI; however, there is still a lack of research on the impact of epigenetic alterations, iatrogenic injuries, environmental pollution, social stress, and unhealthy lifestyles on the pathogenesis of POI.

**Conclusion:**

The factors illustrated here represent potential future directions for POI etiology research and warrant more attention from researchers.

## 1 INTRODUCTION

Premature Ovarian Insufficiency (POI), also known as primary ovarian insufficiency or premature ovarian failure, is one of the most common reproductive endocrine disorders in women of childbearing age [1, 2]. POI is characterized by the development of ovarian hypofunction in women before the age of 40, which occurs in approximately 3.5% female population [1, 3]. POI not only leads to infertility, but also increases the risk of a variety of chronic diseases, such as cardiovascular diseases [4], skeletal fragility [5], and cognitive impairment [6], leading to increased all-cause mortality [7]. Therefore, the study of etiology is of great significance for early warning, diagnosis, and intervention of POI.

Specifically, the occurrence of POI is a complex and multifaceted process, which derives from the diminished ovarian reserve pool and the accelerated ovarian follicle depletion [8, 9]. Recognized risk factors include genetic variation [10-12], epigenetic alterations [13-15], autoimmune diseases [16-19], and the inability to recover following medical procedures, such as ovarian surgery, chemotherapy [20, 21], *etc*. Unfortunately, despite technological advances, particularly in the development of high-throughput sequencing, the POI etiology research has developed slowly, and the cause of the POI is still unknown in the majority of cases [22]. Recently, there has been an increasing amount of research dedicated to the pathogenesis of POI, and numerous studies have been published on the etiology of this disease [23-26]. However, there is currently a lack of systematic study on the development trend in this area.

Bibliometrics and visualization have been considered crucial to evaluating scientific research [27, 28]. This observation is pertinent to the current state of affairs, wherein large amounts of data are being transferred. Bibliometrics is also employed in various fields to assess the quantitative and qualitative nature of the knowledge base [29-33]. Numerous scientists have used this strategy to assess their respective research domains, acquiring vital information, such as the distribution, contribution, and collaboration of countries/regions, institutions, journals, and authors in certain fields [34-39]. Therefore, bibliometrics can facilitate the rapid understanding of a particular field, including research hotspots and evolving trends in this field, and promote research innovation.

This study intended to conduct a bibliometric analysis of publications related to the etiology of POI in the Web of Science database. To achieve this objective, a quantitative method was employed to conduct a bibliometric analysis of the published literature. This study has utilized VOSviewer, bibliometric.com, and Microsoft Excel 2019 for bibliometric analysis. To the best of our knowledge, this is the first bibliometric study to assess research trends in the etiology of POI. By recognizing the key research sites for the etiology of POI, the findings of this study can contribute to the creation of national and institutional research initiatives. Furthermore, the visualization data or evidence can be used to identify potential future research paths and collaborative relationships, promoting the rapid development of POI etiology research.

## 2 MATERIALS AND METHODS

### 2.1 Data Source and Search Strategy

The data used in our study were taken from the Web of Science Core Collection (WoSCC). We searched for relevant literature on POI, published between 1994 and 2023 by using the Science Citation Index Expanded (SCI-E) database of the WoSCC. The following was the retrieval strategy: “((((TS=(etiology)) OR TS=(aetiology)) OR TS=(pathogenesis)) OR TS=(pathology) and ((TS= (premature ovarian insufficiency)) OR TS=(primary ovarian insufficiency)) OR TS=(premature ovarian failure))”. The search and download of data were completed on 31^st^ December, 2023. The document type was limited to research articles. It is important to note that we did not include articles focused on diminished ovarian reserve. Subsequently, we excluded the literature including review articles, proceeding papers, meeting abstracts, book chapters, notes, and editorial material. The search language used was English, and the year of publication was limited to the period from 1994 to 2023. The study only included articles from the Web of Science Core Collection database and, therefore, did not include articles from medRxiv and clinicalTrials.gov. In addition, 28 articles published by predatory journals were also excluded. At the end of the screening process, we obtained a total of 456 papers. The flowchart is shown in Fig. (**1**).

### 2.2 Data Analysis Methods

All the data were exported into VOSviewer (version 1.6.18, Leiden University, Leiden, Netherlands) for further analysis, which could generate map and cluster visualization [36, 40-42]. The online bibliometric analysis platform, available at https://bibliometric.com/app, was used to calculate the publications of countries/regions, institutions, journals, and authors related to POI.

## 3 RESULTS

### 3.1 Analysis of Top Productive Countries/Regions

Based on the search strategy described previously, we obtained a total of 726 papers. Of these records, there were 508 (69.97%) research articles, 174 (23.97%) review articles, 24 (3.31%) proceedings papers, 11 (1.52%) meeting abstracts, 6 (0.83%) editorial materials, 2 (0.28%) book chapters, and 1 (0.14%) note (Fig. **2A**). Subsequently, we excluded 9 articles published before 1994 and then excluded 10 articles in French, 2 articles in German, 1 article in Polish, 1 article in Slovenian, and 1 article in Spanish. Specifically, research articles were derived from a total of 57 countries/regions. Fig. (**2B**) presents a map of the world based on the total number of articles contributed by each country, with different colors representing different volumes of articles. As can be seen, China and the USA contributed the highest number of publications, with more than 120 publications marked in red. However, the regions of northern Europe, Eastern Europe, and Africa all had fewer than 7 publications, suggesting these regions to have great potential in this field of research.

We also analyzed the administrative region sources of published articles in China and the USA. We used heat maps to reflect the research enthusiasm and contribution of different regions to this field. The analysis revealed that although POI-related cases were reported in most regions of the two countries, most of the reports originated from economically developed coastal areas, such as Shandong, Jiangsu, and Guangdong provinces in China (Fig. **3A**), and California, New York, and Maryland in the USA (Fig. **3B**). Whether the differences in the above trends have been due to more research funding in the economically developed regions or whether the incidence of POI has been exacerbated by environmental pollution and psychological stress, future environmental and psychological studies are needed to confirm the results.

Table **1** lists the top 10 countries/regions that have published articles on POI etiology. It can be seen that China ranked first among the countries in terms of total publications, with 141 research articles published in the last 30 years, representing 30.92% of the retrieved articles, suggesting that the etiology research of POI is relatively advanced and fruitful in China. The United States came in second place with 125 publications, accounting for 27.41% of the total publications. France was found to be in the third place with 53 publications. These three countries accounted for 69.95% of all articles consulted, indicating these countries to be among the world leaders in this field.

### 3.2 Trend in the Annual Evolution of Publications

The annual publications on the etiology research of POI are shown in Fig. (**4A**). A gradual upward trend can be seen in the total number of annual publications. Between 1994 and 1999, the number of relevant publications ranged from 1 to 7, a relatively small number. Between 2000 and 2006, the production of relevant research increased slowly, with an annual number of publications being 10 or less. Since then, the production has increased steadily, especially in the past four years from 2020 to 2023, where the number of relevant publications increased rapidly, with an annual production of 34 articles. Actually, the number of publications in the last four years has represented 29.83% of the total number of publications. Also, the number of publications from the USA was in first place. This indicates that the USA was one of the first countries to start research in this area. Between 1994 and 2010, fewer articles were published from China. Nevertheless, the number of publications from Chinese researchers has increased rapidly since 2011. Moreover, China has ranked first in the number of publications in this field since 2019. These observations suggest that although China is a latecomer, they have developed rapidly in recent years and have become a mainstay in the field. In addition to China and the United States, the number of publications in other countries/regions has also been found to be increasing year by year. Therefore, the etiology research of POI has gradually become a hotspot in scientific exploration.

Fig. (**4B**) shows the trend of the annual publications of the top 10 countries/regions over the 30-year period from 1994 to 2023. It can be seen that the United States has published in this field since 1995, almost every subsequent year.

### 3.3 Contributions of Journals and Funding Agencies

We have sorted the journals published in this field according to the number of publications. Table **2** lists the top 10 journals in terms of publication volume from 1994 to 2023. As we can see, these 10 journals have published 133 articles, representing 29.17% of all publications. The most productive journal has been Human Reproduction with 33 articles, representing 7.24% of the total articles retrieved. This has been followed by Fertility and Sterility and the Journal of Clinical Endocrinology and Metabolism, both with 17 publications, representing 3.73%, respectively. These three journals have been top journals in reproductive medicine with high academic standards, indicating the etiology research of POI to be a major concern for reproductive medicine scientists.

A summary of the top 10 most active funding agencies is shown in Table **3**. It can be seen that the National Natural Science Foundation of China supported the largest number of publications with 77 publications (14.98%). This was followed by the National Institutes of Health (NIH) and the United States Department of Health and Human Services with 90 publications (17.51%). A total of 258 articles supported by these top 10 foundations were published, representing 56.58% of all publications. It is noteworthy that among the top 10 foundations, six of these were from China, and three of them were from the USA. This demonstrates the Chinese and American foundations to attach great importance to exploring the etiology or pathogenesis of POI in order to facilitate better treatment options.

### 3.4 Analysis of Influential Authors and Institutions

Table **4** lists the top 10 authors in terms of publication volume in the field of the etiology research of POI. They have published a total of 135 articles, accounting for 29.61% of all papers. Among them, Qin Yingying from China topped the list with 30 articles and 95 citations. She was followed by Chen Zi-Jiang and Zhao Shidou from China. It can be seen that among the top 10 authors in terms of publication volume, six have been from China, indicating Chinese scholars to have a strong desire for exploration in this field and to make some big achievements. In addition, we have calculated the H-index for each author individually during the past 5 years, which reflects the author's influence on the research frontier. However, it is worth noting that several authors in the most productive list have been from the same organization. Although the number of patients reported has been different, there is still a possibility that some patient data could have been shared.

The top 10 institutions with the highest number of publications related to the etiology of POI are listed in Table **5**. Among them, Shandong University ranked first in terms of the number of publications as well as the number of citations. Shandong University achieved a total of 87 articles and 235 citations. This was followed by Shanghai Jiao Tong University and Fudan University from China, with 33 and 24 publications, respectively, and the number of citations was 95 and 22, respectively. The above data indicate that these institutions have played an important role in this field.

### 3.5 Visualization of Research Trend and Evolution Analysis

Firstly, we classified the articles based on cohort types, and we have found most of the articles to be based on studies on patients with non-syndromic POI (Fig. **5A**). Then, for classifying based on the field of studies, we have found the genetic etiology studies to be the most prevalent, followed by immune and epigenetic etiology studies. Studies related to ovarian surgery and chemotherapeutic drugs were less prevalent. Additionally, studies on the environment, lifestyle habits, and mouse models were classified among other classifications (Fig. **5B**).

Keywords are short words or phrases that describe an article's main content. Visualization analysis of keywords can effectively show the hot spots and trends in the field and provide researchers with a large amount of usable information. Based on the keywords provided by authors in the article, a total of 219 keywords were extracted from 456 articles. We selected 113 keywords with more than 5 occurrence times to generate a word cloud map, in which the distribution of hotspots in the whole field could be seen intuitively. As shown in Fig. (**6**), “mutation”, “genetics”, “variants” and “fMR1” were centered in the density map, which indicated them to be the most popular elements.

Title and abstract field terms with more than 10 occurrence times in all retrieved publications were analyzed *via* VOSviewer. After the removal of duplicate or invalid terms, there were 174 terms that reached this threshold in this field, and they were thus divided into four clusters and colored differently. As shown in Fig. (**7A**), the red cluster focused on clinical phenotype and treatment; “FSH”, “menopause”, “chromosomal abnormality”, “treatment”, and other keywords can be seen. The green cluster was related to the pathogenic mechanism and contained keywords of “ovarian development”, “autoimmunity”, “cell”, and “pathway”. The yellow cluster mainly described fragile X syndrome, containing “disorder”, “FMR1” “premutation”, and “CGG repeat” as keywords. The fourth cluster was colored in blue and the terms were related to the causative gene. The most striking terms were “mutation”, “variation”, “whole exome sequencing”, and “genetic”. These results demonstrated the most prominent fields in the etiology research of POI to include 4 directions during the past 30 years.

Fig. (**7B**) shows a network visualization map of the terms overlaid with the occurrence year. The color of each node corresponds to the average year they appeared in the 456 related publications. The terms in blue appeared earlier, and those in yellow and red appeared later. Before 2012, most studies focused on the phenotype of POI. Thereafter, more attention has been paid to the development process of ovarian follicles and genetic pathogenesis. Of note, the latest trends showed that “whole exome sequencing”, “pathogenic variant”, and ‘meiosis’ would be concerned more widely in the future.

## 4 DISCUSSION

Bibliometrics is a study that focuses on the structure of literature distribution, quantitative relationships, and patterns of change using statistical techniques [43-47]. Therefore, bibliometric analysis is an excellent method that provides researchers with a systematic and intuitive structure of knowledge and helps researchers understand the general trends in their fields of study [48-52]. Researchers have published a large number of articles on the etiology of POI over the past 30 years [23, 24, 53, 54], but there are no relevant bibliometric studies to summarize them. VOSviewer and bibliometric.com are bibliometric platforms that allow for a more visual presentation of statistical results [55, 56]. In this study, we used VOSviewer and bibliometric.com to analyze the publication years, countries, institutions, authors, journals, funds, and keywords from the literature related to the etiology of POI from 1994 to 2023. To our knowledge, this is the first bibliometric study to evaluate and visualize research in the field of the etiology of POI. Our results obtained from the bibliometric analysis could help researchers quickly and deeply explore the evolution of topics, major research dimensions, and promising research directions in the field of POI etiology.

The analysis of the trend in the annual evolution of publications has revealed the number of publications in the etiology research of POI to be increasing year by year, especially in the last four years. It means that researchers have achieved a lot of breakthroughs in different aspects of POI etiology. However, the analysis of countries/regions has shown the publications to be mainly derived from regions of Asia, North America, and Europe. Therefore, clinical guidelines or recommendations for the management of POI published in recent years may be more applicable to Asian and Caucasian populations [57-59]. Additionally, it is noteworthy that China has contributed the highest number of publications in this field. Although studies have found the USA to be the country with the most POI-related publications [42], our results have suggested China to be the leader in POI etiology research. The data suggest that Chinese scientists have initiated to address the increasing prevalence of infertility and the reproductive aging of women of childbearing age [60, 10]. Enhancing international cooperation could undoubtedly eliminate academic barriers and promote the development of research on the etiology of POI.

Our current study has found research on the causes of POI in China and the United States to be significantly more extensive compared to other countries worldwide. Previous reports have indicated POI to affect 1% of women [61], but recent studies have shown the prevalence of POI to be increasing, which now stands at 3.5% [1]. Studies have also shown differences in POI occurrence based on ethnicity, with higher rates among Caucasian, African American, and Hispanic women [62]. For instance, the prevalence rate in the United States has been found to be 10.9% [63], while in Canada and Sweden, it has been found to be 13.1% [64] and 13% [65], respectively. In contrast, the prevalence rate has been found to be 2.41% in Korea [66], 2.8% in China [67], and 3.5% in Iran [68]. The higher incidence of POI in the United States has garnered more attention from researchers, while regions with early childbearing, such as Africa [69-71] and Russia [72-74], have received less focus on POI etiology. Although young women in the aforementioned areas typically complete their childbearing before the onset of POI, it is important to raise awareness and enhance screening in these regions due to the long-term health effects of POI [75, 76]. In China, the delayed birth window [77], declining fertility rate [78], and rising infertility incidence [60], in recent years, emphasize the impact of POI on fertility. This situation may likely lead to increased attention and research interest from the government and researchers in reproductive health, promoting the research on the etiology of POI.

The present analysis has revealed more than 2500 researchers to participate in the research concerning the etiology of POI. At least 7 articles have been published by each of the top 10 most productive authors. Among them, Qin Yingying from Shandong University has contributed the most articles, followed by Chen Zi-Jiang and Zhao Shidou. Of note, the above three authors have all been from Shandong University, showing the dominating role of the group of Shandong University in this field. 6 institutions in the top 10 most productive organizations have been found to be from China, and this indicates its leadership in the field. The institution that contributed the most publications in this field was Shandong University. USA has been found to include several of the most prestigious institutions, which may partly explain why it has maintained its leadership in the study of POI etiology. Additionally, the list has contained one French institution and one Italian institution, and the other countries have not been found to involve an institution in the top 10 yet. Therefore, other countries’ researchers should seek collaboration with Chinese and American research groups to promote technological breakthroughs in this field.

Journal indicators obtained from the bibliometric analysis can provide a reliable reference for researchers to search for documents or submit manuscripts [79]. The results have shown Reproductive Medicine, Human Reproduction, and Fertility and Sterility, as the most productive journals involved in the etiology of POI. These journals can provide researchers with reliable references for relevant research or further study. According to the 2022 JCR standards, among the top 10 most productive journals, 4 were ranked Q1, and 5 were ranked Q2, suggesting that these journals have a strong academic performance in POI etiology research, publishing high-quality studies with compelling and proven results.

Keywords reflect the core themes and main contents of publications [80]. Therefore, analyzing the keywords in different ways helps us to better understand and grasp the trends and research hotspots in the research field [81]. By analyzing the keywords given by the authors *via* VOSviewer, we found “mutation”, “genetics”, “fMR1”, “GDF9”, “FOXL2”, “oocyte”, and “mouse model” to be the most frequently appearing keywords. This suggests that genetic factors are the focal point of POI etiologic research. The interest in the genetic causes of POI is not coincidental. This may be largely due to the clinical finding that POI has a significant genetic predisposition. This is supported by the fact that familial POI accounts for 15% of all cases [82]. Additionally, about 50% of menopause age is determined by genetic factors [83], and daughters of patients with early menopause have a six-fold increased risk of early menopause and an eight-fold increased risk of POI [84, 85]. All of these clinical manifestations suggest that genetic factors play an important role in POI.

Terms analysis demonstrated clinical phenotype and treatment, causative gene, fragile X syndrome, and pathogenic mechanism to be the four most important research fields related to POI etiologic research. Temporal distribution analysis has revealed early studies to mainly focus on the population, chromosomal abnormality, and menopause (mainly derived from 2012), indicating that studies began to focus on the phenotype of POI before 2014; this was also a period of accumulation of POI research. The first conversion occurred in 2014 and lasted for two years. The theme terms were related to mechanisms of ovarian follicle development in this stage. After 2016, the concept of genetics and variation began to explode. From the evolution of the terms analysis from 2012 to 2016, concepts related to POI etiologic research have evolved from phenotype to monogenic mechanisms [20]. The second conversion occurred in 2018 and has continued into the present. This evolution can be attributed to the advancement of high throughput sequencing and CRISPR-Cas9 techniques. In the early days, only clinical descriptive analyses or *in vitro* functional experimental studies of de novo variants in known genes could be carried out. With the commercialization of high-throughput sequencing and CRISPR-Cas9 techniques, especially the emergence of whole exome sequencing, numerous causative genes and candidate genes have been identified [53, 86]. Clinicians should provide the POI patients with an option to screen the genetic etiology. High-throughput screening measures and massive data analytics technologies can be a hotspot for POI etiological research in the future.

Over the past 30 years, research on the etiology of POI has made significant progress. The studies have indicated ovarian reserve deficiency and accelerated follicular depletion to be the primary pathogeneses of POI. In the future, large-sample cohort studies [87, 88], long fragment sequencing technology [89, 90], and spatial transcriptome sequencing [91-93] may reveal new evolutionary processes of POI. Currently, genetic studies have identified nearly 100 genes associated with POI [94], while immunological studies have uncovered the relationship between autoantibodies, autoimmune diseases, and the pathogenesis of POI [95]. However, there is still a lack of research on the impact of epigenetic alterations, iatrogenic injuries, environmental pollution, social stress, and unhealthy lifestyles on the pathogenesis of POI. These factors represent potential future directions for POI etiology research and warrant more attention from researchers.

## CONCLUSION

In summary, there is a growing interest in research related to the pathogenesis of POI. Research publications on POI etiology are increasing rapidly, and the research trends in this field are also changing. This study has revealed the top researchers and institutions worldwide to be involved in POI etiology research. Human Reproduction has been found to be the most productive journal, and Qin Yingying the most influential author. Genetic pathogenic factors and mechanisms have been found to be hot research topics. Whole genome sequencing and environmental causative factors may be novel future research directions, warranting international cooperation in this field.

## Figures and Tables

**Fig. (1):**
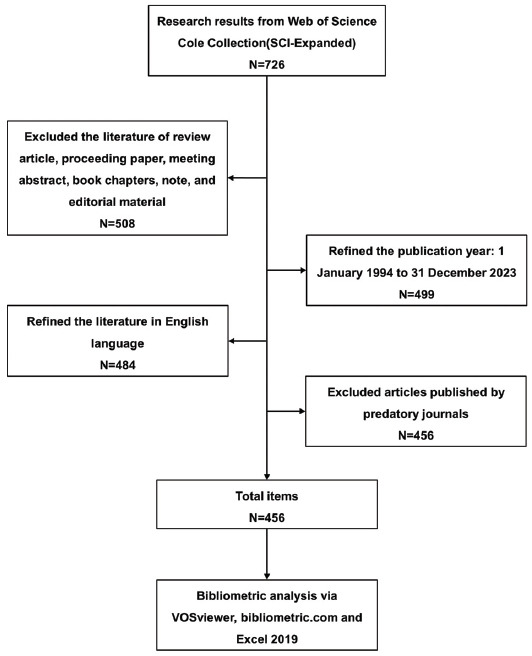
Flowchart of data collection.

**Fig. (2A):**
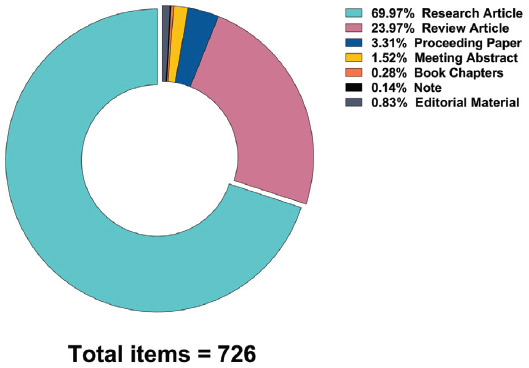
Proportion of publication types related to POI etiology research.

**Fig. (2B):**
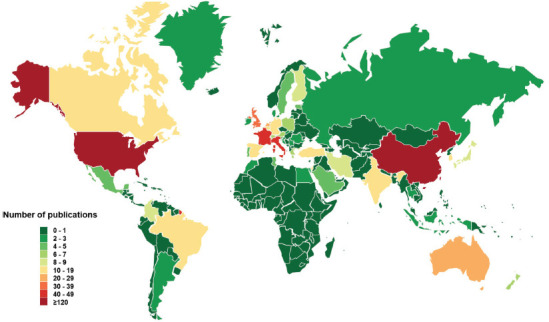
A world map indicating the distribution of countries/regions based on the number of publications related to POI etiology research.

**Fig. (3A):**
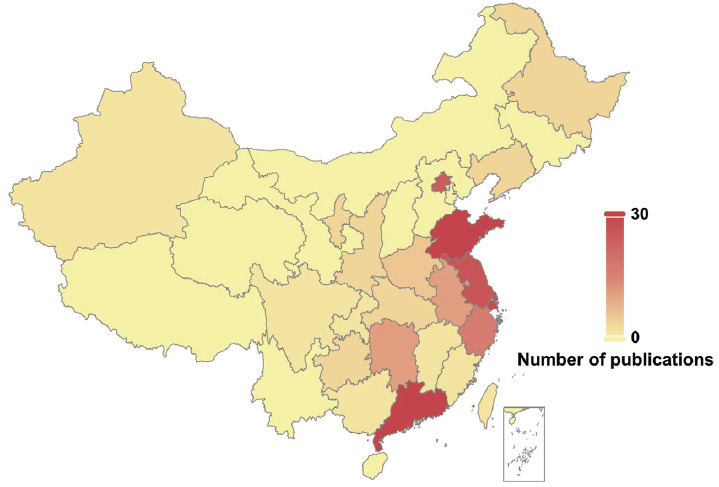
A heat map demonstrating the distribution of Chinese regions based on the number of publications.

**Fig. (3B):**
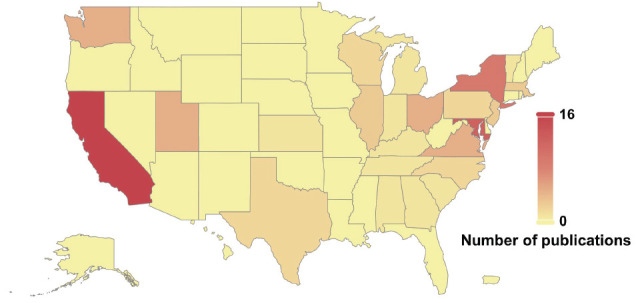
A heat map demonstrating the distribution of the USA regions based on the number of publications.

**Fig. (4A):**
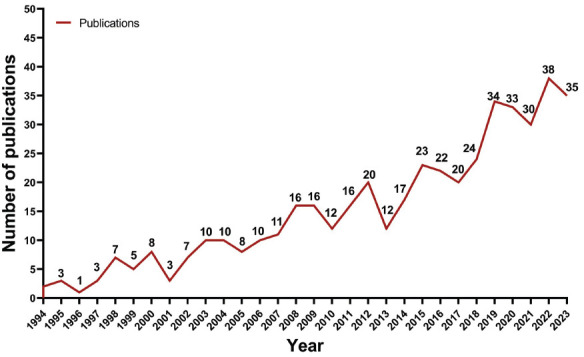
Development of the number of articles from 1994 to 2023.

**Fig. (4B):**
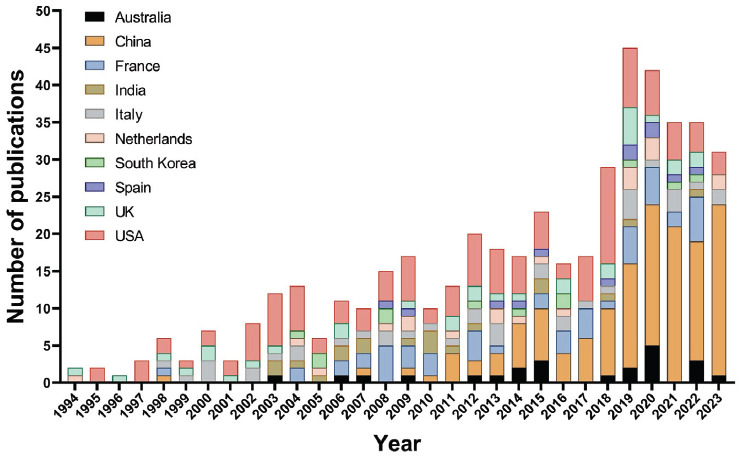
Trend of the annual publications in the top 10 countries/regions.

**Fig. (5A):**
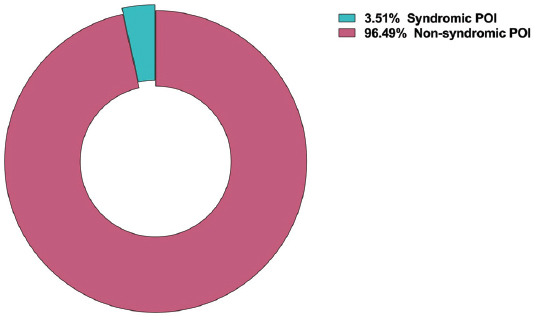
Map of the study population related to POI etiology research.

**Fig. (5B):**
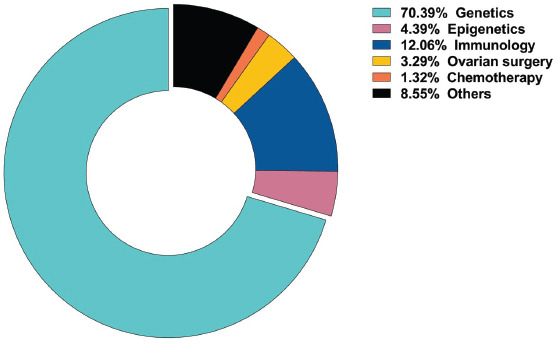
Map of the field of study related to POI etiology research.

**Fig. (6):**
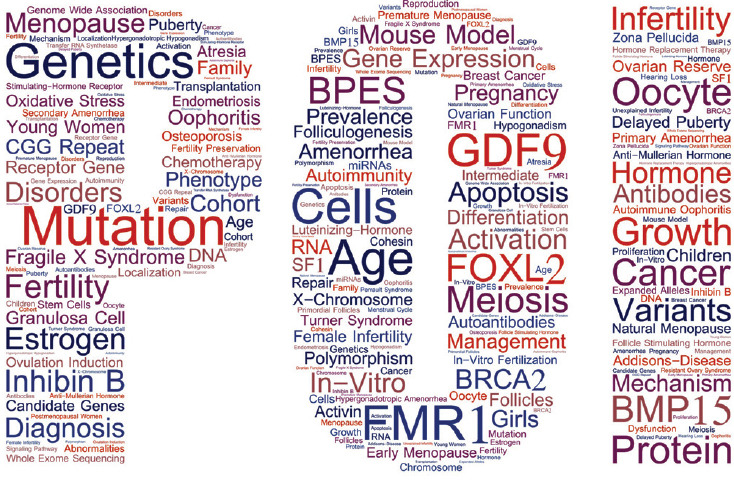
Map of the keywords cloud.

**Fig. (7A):**
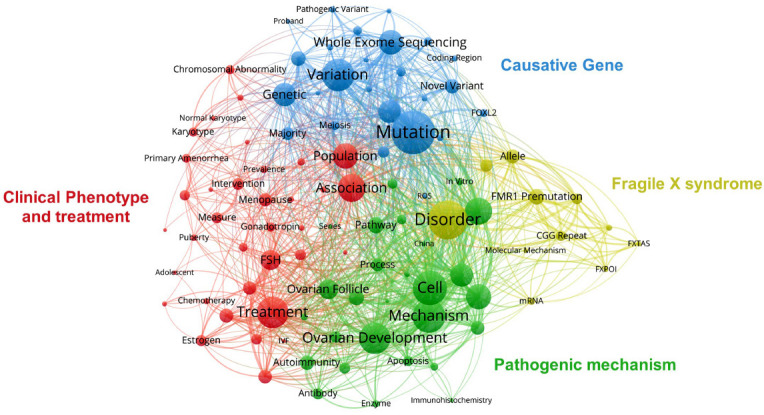
Network visualization mapping of the terms.

**Fig. (7B):**
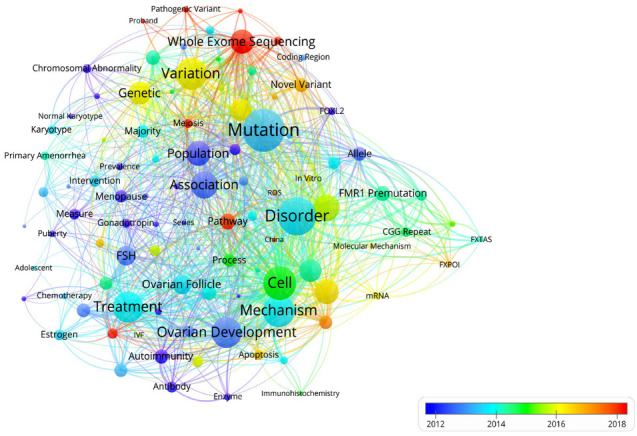
Overlay visualization mapping of the terms.

**Table 1:** The top 10 countries /regions with the highest productivity.

**Rank**	**Country**	**No. of Publications**	**Percentage (%)**
1	China	141	30.92
2	United States	125	27.41
3	France	53	11.62
4	Italy	39	8.55
5	UK	28	6.14
6	Australia	23	5.04
7	Netherlands	20	4.39
8	India	19	4.17
9	Spain	13	2.85
10	South Korea	12	2.63

**Table 2:** List of the top 10 journals with the largest number of publications.

**Rank**	**Journal**	**2022 Impact Factor**	**2022 JCR**	**No. of Publications**	**Percentage (%)**
1	Human Reproduction	6.100	Q1	33	7.24
2	Fertility and Sterility	6.700	Q1	17	3.73
3	Journal of Clinical Endocrinology & Metabolism	5.800	Q1	17	3.73
4	Gynecological Endocrinology	2.000	Q4	11	2.41
5	Journal Of Assisted Reproduction And Genetics	3.100	Q2	11	2.41
6	Plos One	3.700	Q2	10	2.19
7	Human Molecular Genetics	3.500	Q1	9	1.97
8	Journal Of Ovarian Research	4.000	Q2	9	1.97
9	Seminars In Reproductive Medicine	2.700	Q2	9	1.97
10	Maturitas	4.900	Q2	7	1.54

**Table 3:** List of the top 10 funding sources.

**Rank**	**Funding 514**	**Country**	**No. of Publications**	**Percentage (%)**
1	National Natural Science Foundation of China	China	77	14.98
2	National Institutes of Health (NIH)	United States	45	8.76
3	United States Department of Health & Human Services	United States	45	8.76
4	Eunice Kennedy Shriver National Institute of Child Health and Human Development	United States	20	4.09
5	National Key Research and Development Program of China	China	18	3.50
6	National Key Research Developmental Program of China	China	17	3.31
7	National Basic Research Program of China	China	11	2.14
8	National Health and Medical Research Council of Australia	Australia	9	1.75
9	National Science Fund For Distinguished Young Scholars	China	8	1.56
10	Taishan Scholars Program For Young Experts Of Shandong Province	China	8	1.56

**Table 4:** List of the top 10 authors with the most publications.

**Rank**	**Author**	**Country**	**No. of Publications**	**Total Citations**	**H-index**
1	Qin Yingying	China	30	95	20
2	Chen Zi-Jiang	China	15	69	24
3	Zhao Shidou	China	15	51	14
4	Touraine Philippe	France	14	47	15
5	Guo ting	China	14	35	9
6	Jiao Xue	China	11	54	7
7	Veitia Reiner Albert	France	10	43	10
8	Laissue Paul	Colombia	9	61	3
9	Wang Jing	China	9	26	1
10	Marozzi Anna	Italy	8	57	1

**Table 5:** List of the top 10 most productive organizations.

**Rank**	**Institution**	**Country**	**No. of Publications**	**Total Citations**
1	Shandong University	China	87	235
2	Shanghai Jiao Tong University	China	33	95
3	Fudan University	China	24	22
4	The University of Utah	United States	23	53
5	Chinese Academy of Sciences	China	22	77
6	CHU rennes	France	22	61
7	Anhui Medical University	China	22	58
8	University of California	United States	21	18
9	University of Milan	Italy	20	140
10	Zhejiang University	China	20	52

## LIST OF ABBREVIATIONS

NIH: National Institutes of Health
POI: Premature Ovarian Insufficiency
WoSCC: Web of Science Core Collection
